# Effect of Metal Ions on the Interaction of Condensed Tannins with Protein

**DOI:** 10.3390/foods12040829

**Published:** 2023-02-15

**Authors:** Liangliang Zhang, Qinhao Guan, He Zhang, Lihua Tang

**Affiliations:** 1 Academy of Advanced Carbon Conversion Technology, Huaqiao University, Xiamen 361021, China; 2 Institute of Chemical Industry of Forest Products, CAF, Key Laboratory of Biomass Energy and Material, Nanjing 210042, China; 3 Co-Innovation Center of Efficient Processing and Utilization of Forest Resources, Nanjing Forestry University, Nanjing 210042, China; 4 Institute of Biomedical Health Technology and Engineering, Shenzhen Bay Laboratory, Shenzhen 518107, China

**Keywords:** condensed tannins, protein, binding reaction, precipitation, metal ions

## Abstract

A quantitative analysis of the precipitate effects of metal ions (Al^3+^, Fe^2+^, Cu^2+^, Zn^2+^) by bovine serum albumin (BSA) on two condensed tannins (CT) from sorghum and plum was presented in this study. The results showed that adding metal ions enhanced the precipitation of proteins by CT, depending on the type and concentration of the metal ions used in the reaction system. The presence of metal ions and precipitation results on the CT–protein complex showed that Al^3+^ and Fe^2+^ had a higher binding ability with CT and a weaker influence on the precipitation of the CT–protein complex than Cu^2+^ and Zn^2+^. However, when the initial reaction solution contained excessive amounts of BSA, the extra addition of metal ions had no significant effect on the amount of BSA precipitation. Reversely, adding Cu^2+^ or Zn^2+^ into the reaction solution increased the amount of precipitated BSA when the amount of CT was excessive. In addition, the amounts of CT from plum, rather than sorghum, generated more protein precipitate in the presence of Cu^2+^ or Zn^2+^, which may be due to the different binding modes between the metal ion and the CT–BSA complex. This study also proposed a model of the interaction between the metal ion and the CT–protein precipitate.

## 1. Introduction

Vegetable tannins are widely distributed in the plant kingdom and used for foods, feeds, and medicines [1]. Tannins are classified into two subgroups: hydrolyzable tannins (HT) and condensed tannins (CT). HT consists of gallic acid, its dimers (hyxahydroxydiphenic acid), and derivatives. Usually, HT is constructed with polyols as the core structure, which is connected to multiple phenolic carboxylic acids via ester bonds. CT exists at a high concentration level in the bark of timber species such as conifers, eucalypts and leguminous hardwoods. Their molecular weights are higher than hydrolyzable tannins, with the highest being 20,000. CT is a mixture of polymers including flavan-3-ol units with different polymerization degrees and mostly hydroxyl substitutions [2]. The molecular skeleton of CT is made of C_6_∙C_3_∙C_6_ [3]. Since the aromatic rings are mainly connected via carbon bonds, these condensed molecules are more stable with less hydrolysis [4]. Moreover, compared to HT, CT is more abundant in trees; it is more insoluble and decomposes slowly [5].

The protein’s precipitate ability is to define the conjugation characteristic of tannins and plant polyphenols [6]. Tannins, as described by Bate-Smith and Swain, are water-soluble phenolic compounds with molecular weights between 500 and 3000 with the ability to precipitate alkaloids, gelatin, and other proteins [7]. More importantly, the tendency of tannins to interact with proteins can influence the appearance and taste of foods and beverages, the function of ecological and agricultural systems, and the discovery and application of new pharmaceutical agents [8]. Therefore, understanding the mechanism of interaction between tannins and proteins would help us to control and exploit the properties of polyphenols in these diverse systems. Generally, the precipitation reaction of proteins by tannins has two steps: (1) the phenolic hydroxyl groups of tannins hydrogen bind with the protein; (2) the tannins form a hydrophobic layer among the protein molecules in a multi-point binding manner, causing these protein molecules to aggregate and precipitate. Therefore, molecular weights’ differences and structural diversities mainly affect the combination of tannins and proteins [9]. Besides, the pH, solvent, metal ions, and temperature of the reaction system would also affect the reaction progress [2].

Dietary tannins can affect animal nutrition and health by enabling the better utilization of feed proteins, generating anthelmintic effects against gastrointestinal nematodes, lowering nitrogenous and methane emissions [10,11]. Tannins may bind with dietary proteins, thus reducing the degradation of these proteins in the rumen, and enhancing the amount of protein available for digestion in the small intestine [12]. Tannins can form soluble or insoluble complexes with proteins, and the tannin–protein interactions are both tannin- and protein-specific [13]. Forage legumes contain diverse types and amounts of CT, which may modify the digestibility of dietary protein and structural carbohydrates in animal feeds [14]. The amount and structure of the tannins and the animal adaptation determine tannins’ nutritional effects [15]. Bovine serum albumin (BSA) is a well-characterized model protein which has been widely used to investigate tannin–protein interactions [16,17,18]. Amongst examinations of tannin–protein interactions by competitive binding, spectroscopic methods have demonstrated that protein structure plays an essential role in complex formations.

Through the ortho-dihydroxyphenolic group(s), tannins can form complexes with metals, especially with transition metals [19]. Tannin–metal interactions play an important role in regulating the nutrients and toxins in the soil for the plants and affect various environmental processes such as the formation of humic substances [20]. The strategy of coordination of tannins with metal ions has been proposed based on the metal coordination properties of the simpler phenolics (such as gallic acid, catechin and methyl gallate), which have been studied for many decades and are well established [21]. It is well known that both CT monomers and oligomers can chelate with metal ions. However, the role of metal ions in tannin–protein interactions has not been clearly established.

It is thought that the difference between the complexation with protein and the metal ion is as follows. The complexation of polyphenols with proteins may be considered as a two-stage process of adsorption and agglutination. In the adsorption stage, on the one hand, the hydrogen bonds between the phenyl groups and the carbonyl groups of peptide linkages are formed [22]. On the other hand, on the complexation with the metal ion, the coordinate bonds between the oxygens of phenolic hydroxyl groups and the metal ion are formed. Coordinate bonds are much more affected than hydrogen bonding by hydroxylation patterns in condensed tannins on the metal complexation. However, there is little known about the effect of metal ions on the CT–protein precipitates, such as their protein binding capability, and the amount of CT and protein in precipitates. It is important to improve our knowledge about this issue to better understand animal nutrition and the ecological role of tannins. Therefore, this study used two pure and chemically defined CTs purified from sorghum seeds and plums. They were used to investigate the correlation reaction between CT, proteins, and metal ions.

Another additive often used in feed production and can react with metal ions is acidifying agents. As one of the environmentally friendly feed additives, acidifying agents have the advantage of being non-resistant, residue-free, and non-toxic compared to traditional feed additives, and have an important role in improving the gastrointestinal conditions of animals and promoting the absorption and utilization of nutrients. In practice, organic and inorganic acids are often used as a combination. Common acidifying agents used in feed additives include citric acid, fumaric acid, vitamin C, and ethylene diamine tetraacetic acid (EDTA). Acidifying agents in the body mainly occur in the complexation reaction with metal elements, thus preventing the formation of insoluble salts that are not easily absorbed, and play a role in promoting the absorption and utilization of metal elements. At the same time, acidifying agents can also release hydrogen ions to lower the pH value in the gut of animals and improve the gastrointestinal environment of animals. Therefore, based on the previous study, four more commonly used feed acidifying agents citric acid, fumaric acid, vitamin C, and EDTA were selected for this study. Tannic acid (TA) and plum CT were used as representatives of hydrolyzable tannin and condensed tannin to study the effect of acidifying agents on the reaction between tannin and metal ions and to better understand the stability of tannin and metal ion complexation.

As a ruminant feed additive, tannin has recently attracted much attention from many researchers due to its potential effects on modulating ruminant performance and vitality; it also mitigates methane emissions. Unfortunately, there is a lack of feasibility studies on the utilization of tannins as feed additives. The primary purposes of this study are: (1) to figure out whether metal ions Cu^2+^, Zn^2+^, Al^3+^, Fe^2+^ could influence the amount of CT–protein precipitates; (2) to determine whether metal ions could affect the amount of CT and protein in the precipitates; (3) to investigate whether the different metal ions have the different effects on the CT–protein precipitates; (4) to find out what the metals’ functions are in the CT–protein precipitation; and (5) to examine the effects of four commonly used acidifying agents in feed on the stability of metal ions and tannin complexes.

## 2. Materials and Methods

Materials. Two CT were purified from *Sorghum bicolor* (Moench) grain and plum according to the method described by Zhang and Lin [23]. TA was purchased from Chicheng Biotech Company (Wufeng, China). Bovine serum albumin (BSA) was purchased from the Sino-Biotechnology Company (Shanghai, China). The BCA protein assay kit was purchased from Shanghai Yise Medical Technology Co., Ltd. (Shanghai, China). BSA solution (0.05 mM) was prepared in pH 4.9 acetate buffer solution. The CT solutions (2 mg/mL) were prepared in 50% methanol. The working solutions of Zn^2+^, Cu^2+^, Al^3+^ and Fe^2+^ (5 mM) were prepared by dissolving ZnF_2_, CuCl_2_∙3H_2_O, AlCl_3_∙6H_2_O, and FeSO_4_∙7H_2_O, respectively, in double-distilled water. Dissolve FeCl_3_ in a 0.1 mol/L solution of HCl to make the solution of Fe^3+^ (1 mM). The 1 mM working solution of tannins was made by dissolving plum CT and TA in a pH 4.9 acetate buffer solution. The 1 mM working solution of acidifying agents was made by dissolving citric acid, vitamin C, fumaric acid, and EDTA in water. All other reagents and solvents were of analytical reagent grade. All aqueous solutions were prepared using fresh double-distilled water.

Precipitation experiment. Briefly, in the condition of low concentration (0.27 mg/mL) of CT, 800 μL of pH 4.9 acetate buffer and 200 μL of 2 mg/mL CT solution were added into tubes, followed by 500 μL of 50 μM BSA solution. In the condition of high concentration (0.67 mg/mL) of CT, 500 μL of pH 4.9 acetate buffer and 500 μL of 2 mg/mL CT solution were added into tubes, followed by 500 μL of 50 μM BSA solution. Then, the solutions were mixed intensively and contained the final amount of 0.4 or 1 mg CT and 0.025 μmol BSA. The tubes were centrifuged at 10,000 rpm for 5 min at 4 °C and both cases observed the precipitation of CT and BSA. All experiments were repeated three times.

Effect of metal ions on the precipitated CT and BSA. To ensure that the amount of BSA in the initial reaction solution exceeded the CT, 600–800 μL of acetate buffer and 200 μL of 2 mg/mL CT solution (pH 4.9) were mixed, then 0–200 μL of 5 mM metal ion solution (Al^3+^, Fe^2+^, Cu^2+^, Zn^2+^) and 500 μL of 50 μM BSA solution were added in sequence. The solution with 0.4 mg of CT, 0.025 μmol of BSA, and 0–1 μmol of metal ions was thoroughly mixed. To ensure that the amount of CT in the initial reaction solution exceeded BSA, 300–500 μL of acetate buffer and 500 μL of 2 mg/mL CT solution (pH 4.9) were mixed, then 0–200 μL of the 5 mM metal ion solution (Al^3+^, Fe^2+^, Cu^2+^, Zn^2+^) and 500 μL of the 50 μM BSA solution were added. The solution with 1 mg of CT, 0.025 μmol of BSA, and 0–1 μmol of metal ions was thoroughly mixed. All experiments were repeated three times. The tubes were centrifuged at 10,000 rpm for 5 min at 4 °C, and the supernatant was collected, and the contents of CT in the supernatant were determined by 1-Butanol-HCl method [24]. The contents of BSA were measured by the BCA method [25]. The CT in precipitate was calculated by the initial amounts of CT in the mixture minus the amount of CT in supernatant. The BSA in precipitate was calculated by the initial amount of BSA in the mixture minus the amount of BSA in supernatant.

Effect of acidifying agents on tannin–metal complex stability. A total of 1000 μL of the acetate buffer solution (pH 4.0) was added to the quartz cuvette to blank the spectrometer. A total of 500 μL of 1 mM tannin solution and 500 μL of 1 mM Fe^3+^ solution was added to the cuvette, mixed well, and their absorbance values at 280 nm were measured. Then, 1 mM of the acidifying agent solution (citric acid, fumaric acid, EDTA, vitamin C) was titrated into the mixed solution. Titration started by adding 2 μL of the 1 mM acidifying agent solution to the cuvette and recording the absorbance values at 280 nm after 2 min of mixing. The titration procedure continued with an increment of 2 μL. The absorbance values at 280 nm were recorded for each titration point until a final volume of 16 μL of the acidifying agent solution was added. Each experiment was repeated at least three times.

Statistical analysis. A one-way ANOVA test was performed on the amount of CT and protein in the precipitates to investigate significant differences between different experimental conditions. The method used to discriminate among the means was Duncan’s multiple range test. The computer program/software used was IBM SPSS Statistics software for Windows, version 19.0.

## 3. Results and Discussion

### 3.1. Precipitation of CT and BSA

**Figure 1:**
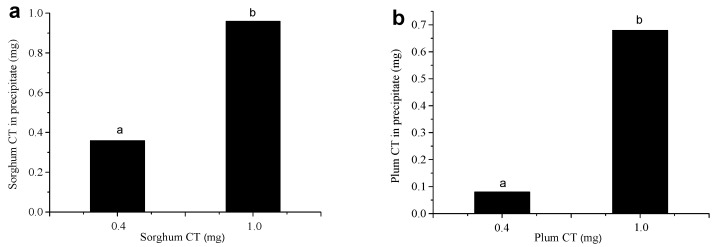
The amount of precipitated sorghum CT (**a**) and plum CT (**b**) by 0.025 μmol BSA in the absence of metal ions at pH 4.9. Means with different lower case on the top of bar are significantly different (*p* < 0.05).

The precipitation experiment showed that both CT and proteins produced precipitation (Figure 1). In the initial solution with 0.4 mg CT and 0.025 μmol BSA, 89.75% sorghum CT was precipitated, while only 20% plum CT was precipitated. When the initial solution contained 1 mg CT and 0.025 μmol BSA, 95.9% sorghum CT and 71.2% BSA were precipitated, whereas only 68% plum CT and 69.98% BSA were precipitated. These results indicated that more sorghum CT could be precipitated by 0.025 μmol BSA compared to plum CT. The precipitation ability of sorghum CT to BSA was more potent than that of plum CT. The binding and precipitation ability of a hydrolyzable tannins compound pentagalloy glucose (PGG) to BSA was studied by Zhang et al. [26]. They indicated that 44.25% PGG was precipitated when BSA was in excess over PGG in the initial solution, while 49.46% PGG was detected when PGG exceeded BSA. This finding is consistent with the report by Hagerman et al. [27]. As observed, CT exhibited stronger binding and precipitation ability to protein than HT.

Moreover, the size of the molecular weight is also a decisive factor affecting the binding of tannins and proteins [28]. Different CTs have different molecular weights due to different polymerization degrees. As the molecular weight increases, the number of phenolic hydroxyl groups bound to the protein will also increase [29,30], thus providing more sites for hydrogens bonding between protein and tannins [31]. As a result, within a specific range, tannins with a high polymerization degree can undergo complexation reactions with more protein [32], which can explain why sorghum and plum CT have different abilities to precipitate protein. In addition, there is a two-way selectivity between proteins and tannins. Plum CT may have a special structure that makes it more capable of binding protein than sorghum CT. The difference in structure is also one of the reasons for this difference. CTs are made of polymers of flavanols [33], and their structure is more complex than hydrolyzable tannins. The chemical structure of CT with higher polymerization degrees is still unclear except for several pentagons and hexamers. Unlike hydrolyzable tannins, the units of CT are connected via C-C bonds, and there are steric hindrances to prevent them from rotating freely. The molecular structure is more rigid and stable [4]. However, proteins with flexible molecular structures are more likely to undergo complexation reactions with tannins to produce precipitation.

### 3.2. Amount of CT in CT–Protein Precipitate

Our previous study [26] indicated that some biologically important metal ions (Al^3+^, Fe^2+^, Cu^2+^, Zn^2+^) positively affect the precipitation of the HT–protein complex and this phenomenon was also observed in the present study for CT–protein precipitate.

**Figure 2:**
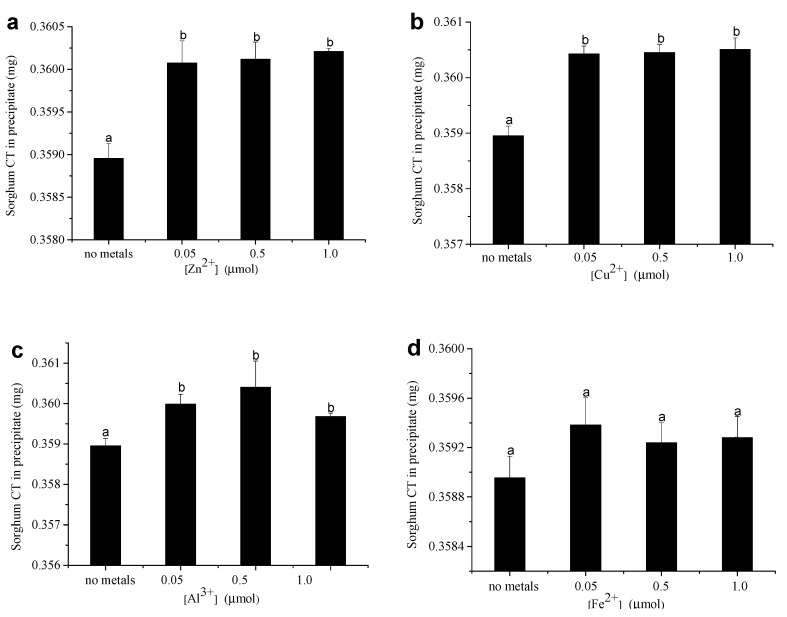
The influence of 0.05–1.0 μmol Zn^2+^ (**a**), Cu^2+^ (**b**), Al^3+^ (**c**), and Fe^2+^ (**d**) on the amount of precipitated sorghum CT by 0.025 μmol BSA in the condition of the reaction solution containing 0.4 mg of sorghum CT. Means with different lower case on the top of bar are significantly different (*p* < 0.05).

Figure 2 shows the effect of metal ions on the amount of CT in the CT–protein precipitates. To make all CT be precipitated by protein, the precipitating experiment was carried out in the condition of the reaction solution containing excessive amounts of BSA (0.4 mg sorghum CT and 0.025 μmol BSA). In the absence of metal ions, 89.75% sorghum CT was precipitated. In the same test conditions, adding 0.05 μmol of Zn^2+^ increased the precipitated sorghum CT from 89.75% to 90.02%. However, there was no significant change in the precipitated sorghum CT when more Zn^2+^ (1 μmol) was added to the solution (Figure 2a). Similar results were observed from the experiment of the sorghum CT–protein precipitation in the presence of Cu^2+^ and Al^3+^. Adding 0.05 μmol Cu^2+^ and Al^3+^ to the solution increased the precipitated sorghum CT from 89.75% to 90.11% and 90.00% (Figure 2b,c). However, the precipitation amount of the sorghum CT did not change significantly when 0.05–1.0 μmol of Fe^2+^ was added to the solution (Figure 2d). When the initial reaction solution contained 1 mg of sorghum CT and 0.025 μmol of BSA, more sorghum CT (95.90%) could be precipitated by the same amount (0.025 μmol) of BSA. Under these experimental conditions, the addition of metal ions had similar influences on the precipitation amount of the sorghum CT when BSA was excessive. In addition to Fe^2+^, other metal ions (Zn^2+^, Cu^2+^, Al^3+^) at a concentration of 0.05 μmol significantly increased the precipitation amount of sorghum CT (Supplemental Figure S1a–d).

**Figure 3:**
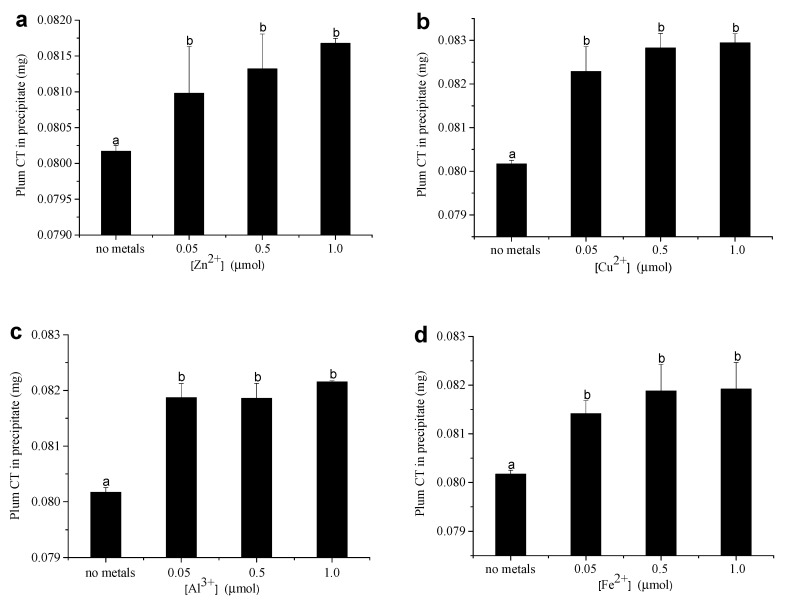
The influence of 0.05–1.0 μmol Zn^2+^ (**a**), Cu^2+^ (**b**), Al^3+^ (**c**), and Fe^2+^ (**d**) on the amount of precipitated plum CT by 0.025 μmol BSA in the condition of reaction solution contains 0.4 mg plum CT. Means with different lower case on the top of bar are significantly different (*p* < 0.05).

The influence of Zn^2+^, Cu^2+^, Al^3+^, and Fe^2+^ on the amount of precipitated plum CT when the initial reaction solution containing 0.4 or 1 mg of plum CT was determined, and the results, are shown in Figure 3. In the absence of metal ions, 20% of plum CT was precipitated when the solution contained 0.4 mg of plum CT and 0.025 μmol of BSA. When the solution contained 1 mg of plum CT and 0.025 μmol of BSA, 68% of plum CT was precipitated. Under the initial solution containing 0.4 mg of plum CT and 0.025 μmol of BSA, adding 0.05 μmol of Zn^2+^ significantly increased the precipitated plum CT from 20% to 20.24%. However, there was no significant change in the amount of precipitated plum CT when more Zn^2+^ (0.5–1.0 μmol) was added to the solution (Figure 3a). Comparable results were also observed in Cu^2+^, Al^3+^, and Fe^2+^. Adding 0.05 μmol of Cu^2+^, Al^3+^, and Fe^2+^ to the solution significantly increased the precipitated plum CT from 20% to 20.57%, 20.47%, and 20.35%, respectively. However, there was no significant change in the amount of precipitated plum CT when 0.5–1.0 μmol of metal ions (Cu^2+^, Al^3+^, Fe^2+^) were added to the solution. Our results indicated that all tested metal ions could significantly increase the amount of precipitated plum CT at 0.05–1.0 μmol (Figure 3b–d). The phenomenon was different when plum CT excessed over BSA in the initial solution. The precipitated plum CT increased from 68% to 68.45% when 0.05 μmol of Zn^2+^ was added (Supplemental Figure S2a). However, there was no significant change in the amount of precipitated plum CT when more Zn^2+^ (0.5–1.0 μmol) was added to the solution. The amount of precipitated plum CT had no significant change when 0.05 μmol of Cu^2+^ was added, whereas the amount of precipitated plum CT increased from 68% to 68.17% and 68.24% when the solution contained 0.5 and 1.0 μmol of Cu^2+^, respectively (Supplemental Figure S2b). These results were also observed in the presence of Al^3+^ under similar experimental conditions. The amount of precipitated plum CT increased from 68% to 68.11% and 68.17% when the solution contained 0.5 and 1.0 μmol of Al^3+^, respectively (Supplemental Figure S2c). However, the precipitation amount of the sorghum CT did not change significantly when Fe^2+^ was added (Supplemental Figure S2d).

### 3.3. Amount of BSA in CT–Protein Precipitate

**Figure 4:**
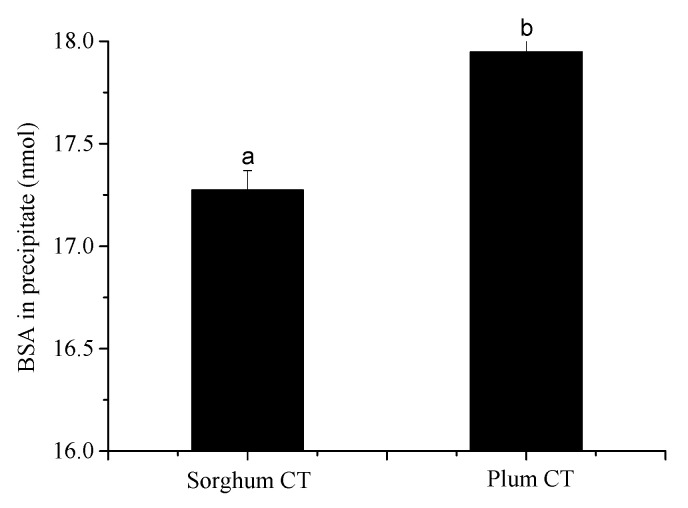
The amount of precipitated BSA by 1 mg of sorghum/plum CT in the absence of metal ions at pH 4.9. Means with different lower case on the top of bar are significantly different (*p* < 0.05).

**Figure 5:**
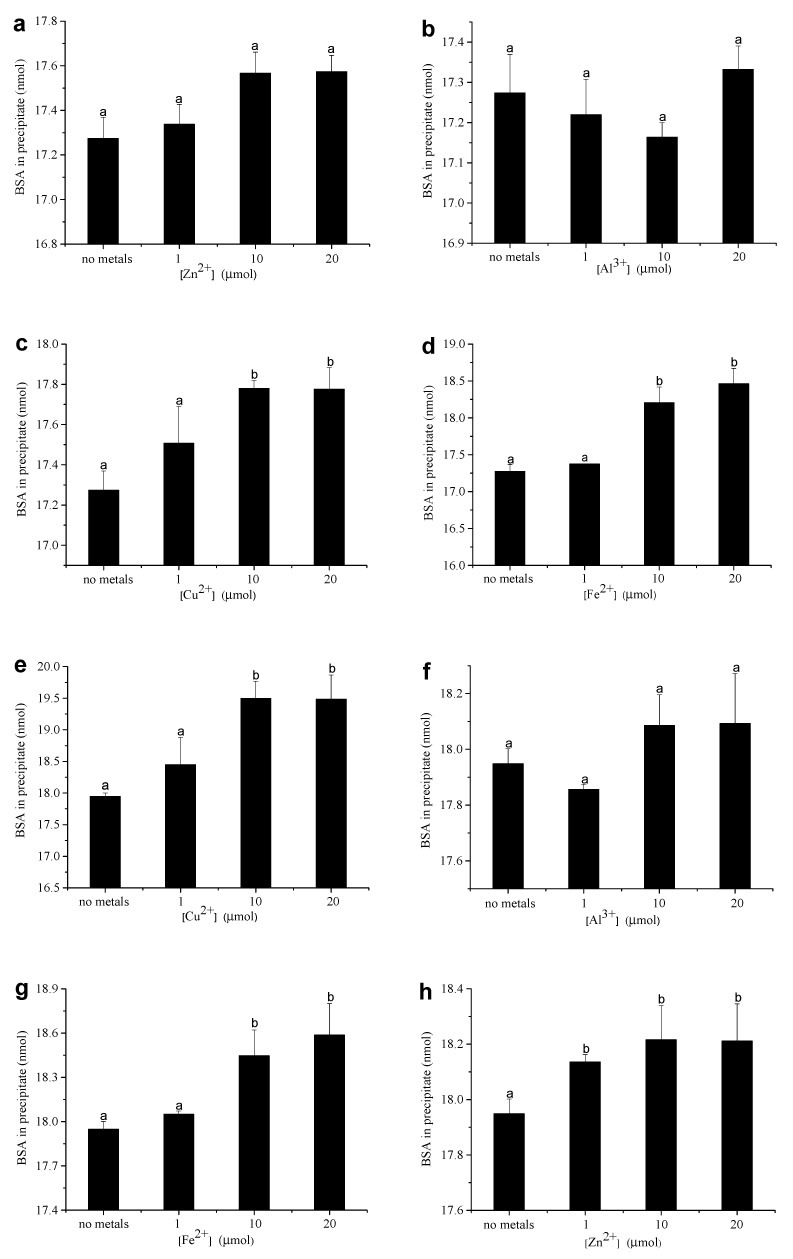
The amount of BSA precipitated by 1 mg of sorghum CT in the presence of 1–20 μmol Zn^2+^ (**a**), Cu^2+^ (**b**), Al^3+^ (**c**), and Fe^2+^ (**d**). The amount of BSA precipitated by 1 mg of plum CT in the presence of 1–20 μmol of Cu^2+^ (**e**), Al^3+^ (**f**), Fe^2+^ (**g**) and Zn^2+^ (**h**). Means with different lower case on the top of bar are significantly different (*p* < 0.05).

Our previous study [26] indicated that metal ions can promote HT–protein precipitation and increase the amount of BSA in the precipitate. To better understand the effect of metal ions on CT–protein precipitation, the influence of metal ions (Al^3+^, Fe^2+^, Cu^2+^, Zn^2+^) on the precipitation amounts of BSA in the CT–BSA precipitates was determined in this study. The precipitation experiment was carried out in the condition of the initial solution containing 1 mg of CT, 0.025 μmol of BSA, and 1–20 μmol of metal ions. Figure 4 shows that the amount of BSA in the precipitates increased with the addition of metal ions (Al^3+^, Fe^2+^, Cu^2+^, Zn^2+^). When the CT–BSA precipitation reaction was run in the absence of metal ions, 71.79% of plum CT and 69.09% of sorghum CT were precipitated by 0.025 μmol of BSA (Figure 4). For sorghum CT, when 1–20 μmol of Zn^2+^ and Al^3+^ were added into the solution, there was no significant change in the precipitated amount of BSA (Figure 5a,b). The amount of precipitated BSA also had no significant change when 1 μmol of Cu^2+^ or Fe^2+^ were added; however, the amount of precipitated BSA increased from 69.09% to 71.12% and 73.85%, respectively, when 20 μmol of Cu^2+^ or Fe^2+^ were added into the solution (Figure 5c,d). The experimental results of the plum CT were not identical to that of sorghum CT. Cu^2+^, Al^3+^, and Fe^2+^ had a similar influence on the amount of BSA precipitated by plum CT and sorghum CT (Figure 5e–g). However, the amount of precipitated BSA increased from 71.79% to 72.54% when 1 μmol of Zn^2+^ was added, and there was no significant change when more (10–20 μmol) Zn^2+^ was added (Figure 5h). The results showed that Cu^2+^ and Fe^2+^ greatly affected the amount of precipitated BSA, while Al^3+^ had the weakest effect on the precipitated amount of BSA. Overall, the amount of precipitated BSA was related to ionic strength.

Figure 5a–h illustrates that the effects of four tested metal ions on the precipitation experiment of two different CTs with BSA were different. The hydrophobic interactions and hydrogen bonding cause CT and protein binding reactions [34]. When metal ions were added to the solution, the metal ions formed coordination bonds with the phenolic hydroxyl groups in the structure of protein and tannins, further enhanced cross-linking between each other, increased the hydrophobic force between tannins and protein, and promoted the production of precipitation [35]. In addition, metal ions formed complexes with tannins in the form of “bridge bonds”. Some metal ions such as Fe^3+^, Cu^2+,^ and Sn^4+^ oxidized phenolic hydroxy groups of tannin molecules into semiquinone structure and formed a protein-tannin complex through mutual polymerization with protein, or the semiquinone structure was further oxidized to quinone, and the protein undergoes a Schiff’s reaction to form a protein-polyphenol complex [22]. Their effects on tannin–protein reactions were special due to differences in different metal ion properties. For example, Al^3+^ enabled proanthocyanin–protein complexes to form a complex and firm reticulate the connection form [36], thus affecting the bio-availability of tannin–protein. When Ca^2+^ had a high concentration, it competed with the protein to bind to tannins.

Compared with hydrolyzable tannins, metal ions have a weaker effect on the complexation and precipitation of condensed tannins with BSA [26]. According to our previous studies [26,37], metal ions exhibit a more substantial binding capacity with hydrolyzable tannins than with condensed tannins, so they are easier to participate in the precipitation reaction of hydrolyzable tannins with BSA. Due to the weak binding ability of metal ions with condensed tannins, fewer metal ions participated in the precipitation reaction of condensed tannins with BSA when the solution contained the same amount of metal ions. These results explain why metal ions had a weaker effect on condensed tannins with BSA precipitation than hydrolyzable tannins with BSA.

### 3.4. Effect of Acidifying Agents on Tannin–Metal Complexation

**Figure 6:**
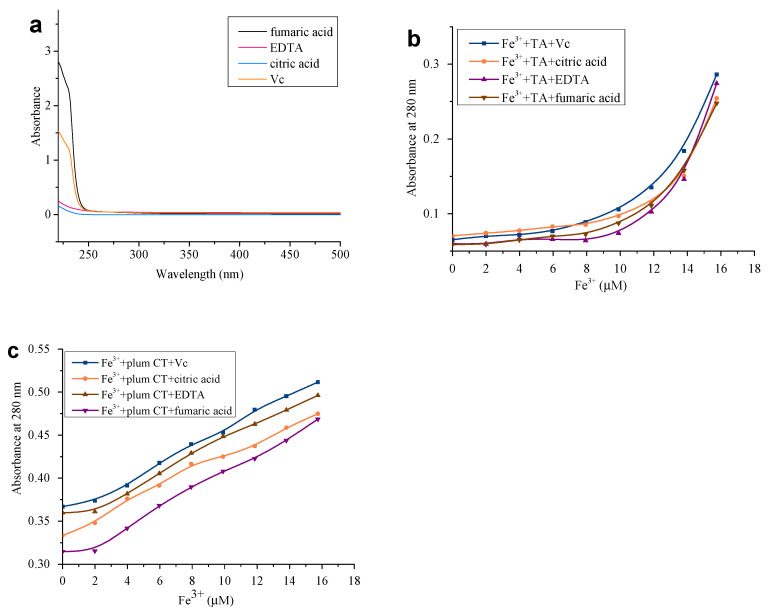
UV-Vis spectra of the four acidifying agents at 190–500 nm (**a**). Effect of acidifying agents on the absorbance values at 280 nm of mixed TA-Fe^3+^ solutions (**b**) and plum tannin–Fe^3+^ solutions (**c**).

From the experimental results, it can be seen that the addition of acidifying agents marginally affects the complexation of tannins and metal ions. The varied results may be due to the different types of acidifying agents and tannins. Figure 6a shows the UV-Vis spectra of the four acidifying agents at 190–500 nm, and it can be seen that they all have no absorption peaks at 280 nm. In comparison, when four acidifying agents were added to the mixture of two different tannins and Fe^3+^, the absorbance values of the mixed solutions at the characteristic absorption wavelength of 280 nm for the tannins began to change (Figure 6b,c). The graphs illustrate that when the acidifying agents were added to the different tannin–Fe^3+^ mixtures, the tannin content of the solutions all gradually increased, indicating that the acidifying agents can react with the metal ions and can displace the tannins from their complex structure to form metal ion-acidifying agent complexes, increasing the tannin content of the mixed solutions. This combined reaction between the acidifier and the metal ion affects the promotion of the tannin precipitated protein by the metal ion. By calculating the ratio of the tannin content displaced by the addition of the acidifier to the tannin content of the initial reaction solution, we can learn that the four acidifiers affect the tannin content in the following order: citric acid > vitamin C > EDTA > fumaric acid. This may be related to the strength of their binding capacity to the metal ions, with the acidifying agent with the stronger binding capacity having a better replacement capacity. Related studies have also shown that citric acid has a more significant binding and replacement capacity for metal ions such as Zn, Cu, and Pb than EDTA [38]. It can also be seen that the absorbance values of the mixed solutions do not change when a small amount of acidifier is added to the Fe^3+^-TA solution until the amount of acidifier added reaches 10 μL and when the absorbance values start to increase significantly. In contrast to the plum CT, the acidifying agent displaces the TA with greater difficulty. Comparing the speed of changes in the absorbance values of the two tannin–metal ion mixtures induced by the addition of the acidifying agent reveals their relationship with the stability of the metal ion complex: TA > plum CT. This difference may be related to the type of tannin—TA belongs to hydrolyzable tannins, which have a stronger binding ability with metal ions, so when an acidifying agent is added to the mixed tannin–metal ion solution, the acidifying dose required to displace the tannin from the hydrolyzable tannin–metal ion complex is greater, and therefore, the complexation between hydrolyzable tannins and metal ions is less affected by the acidifying agent and is more stable.

It is clear from the experimental results that although the acidifying agent affects the stability between tannins and metal ions to some extent, it does not change the reaction trend of metal ions, thus promoting the tannin precipitation of proteins.

## 4. Conclusions

In this work, we studied the effect of metal ions on condensed tannin precipitation complex proteins with representatives of sorghum tannins and plum tannins. We set two scenarios: protein excess and condensed tannin excess. Our experimental results revealed that adding the four metal ions affected the precipitation of CT and BSA, especially Cu^2+^ and Fe^2+^. The addition of metal ions significantly strengthened the ability of condensed tannins to precipitate proteins. Cu^2+^ and Fe^2+^ greatly affected the amount of precipitated BSA, while Al^3+^ had the weakest effect on the precipitated amount of BSA. The amount of precipitated BSA was related to ionic strength. Due to the structure of condensed tannins and the way they combined with metal ions, we speculated that it probably referred to two reasons: (1) when metal ions were added, they combined with condensed tannins in the form of “bridge bonds”, thereby binding more proteins; (2) the addition of metal ions made the condensed tannins combine with themselves to form a network structure, which is more conducive to the binding of proteins. It can be concluded from the experiments of acidifying agents that adding acidifying agents (citric acid, vitamin C, EDTA, and fumaric acid) will destroy the complexation between tannin–metal ions, and the acidifying agents will combine with metal ions in a competitive form to displace the tannins. The ability of four acidifying agents to complex metal ions decreased in order: citric acid > vitamin C > EDTA > fumaric acid; the stability of tannin complexation with metal ions decreased in order: TA > plum tannin, i.e., hydrolyzable tannin complexation stability is stronger than condensed tannin.

In recent years, researchers have paid more and more attention to finding natural plant-derived feed additives. As the natural feed additive for preventing diarrhea and improving intestinal health, tannins have higher safety and a great application prospect in the partial or complete replacement of ZnO and feed antibiotics. They are significant to the efficient and safe production of livestock and poultry feed. The study of the reaction between tannins of different structures and proteins and the possible effects of metal ions can improve the development and utilization of tannins as natural feed additives. It also promotes related research on the tannin precipitation of proteins in animals and plants. Furthermore, it helps to establish a quantitative analysis method to study the effect of metal ions on the precipitation of proteins with tannins. The quantitative analysis methods and theoretical basis for tannins are beneficial in animal nutrition research and feed processing.

## Data Availability

Not applicable.

## Supplementary Materials

The following supporting information can be downloaded at: https://www.mdpi.com/article/10.3390/foods12040829/s1. Supplemental Figure S1. The influence of Zn^2+^ (a), Cu^2+^ (b), Al^3+^ (c), and Fe^2+^ (d) (0.05–1.0 μmol) on the amount of precipitated sorghum CT by 0.025 μmol of BSA in the condition of the reaction solution containing 1 mg of sorghum CT. Supplemental Figure S2. The influence of Zn^2+^ (a), Cu^2+^ (b), Al^3+^ (c), and Fe^2+^ (d) (0.05–1.0 μmol) on the amount of precipitated plum CT by 0.025 μmol of BSA in the condition of the reaction solution containing 1 mg of plum CT.
