# Supplementary material for: Effect of Metal Ions on the Interaction of Condensed Tannins with Protein
Source: Foods. 2023 Feb 15;12(4):829. doi: 10.3390/foods12040829 (PMC9957110; doi:10.3390/foods12040829)
Supplement: Supplementary file 1 [file foods-12-00829-s001.zip › foods-2213041-supplementary.pdf]

Supplemental Figure S1. The influence of Zn^{2+} (a), Cu^{2+} (b), Al^{3+} (c), and Fe^{2+} (d) (0.05-1.0 μmol) on the amount of precipitated sorghum CT by 0.025 μmol BSA in the condition of reaction solution contains 1 mg sorghum CT. Means with different lower case on the top of bar are significantly different ($P < 0.05$).

Supplemental Figure S2. The influence of Zn^{2+} (a), Cu^{2+} (b), Al^{3+} (c), and Fe^{2+} (d) (0.05-1.0 μmol) on the amount of precipitated plum CT by 0.025 μmol BSA in the condition of reaction solution contains 1 mg plum CT. Means with different lower case on the top of bar are significantly different ($P < 0.05$).

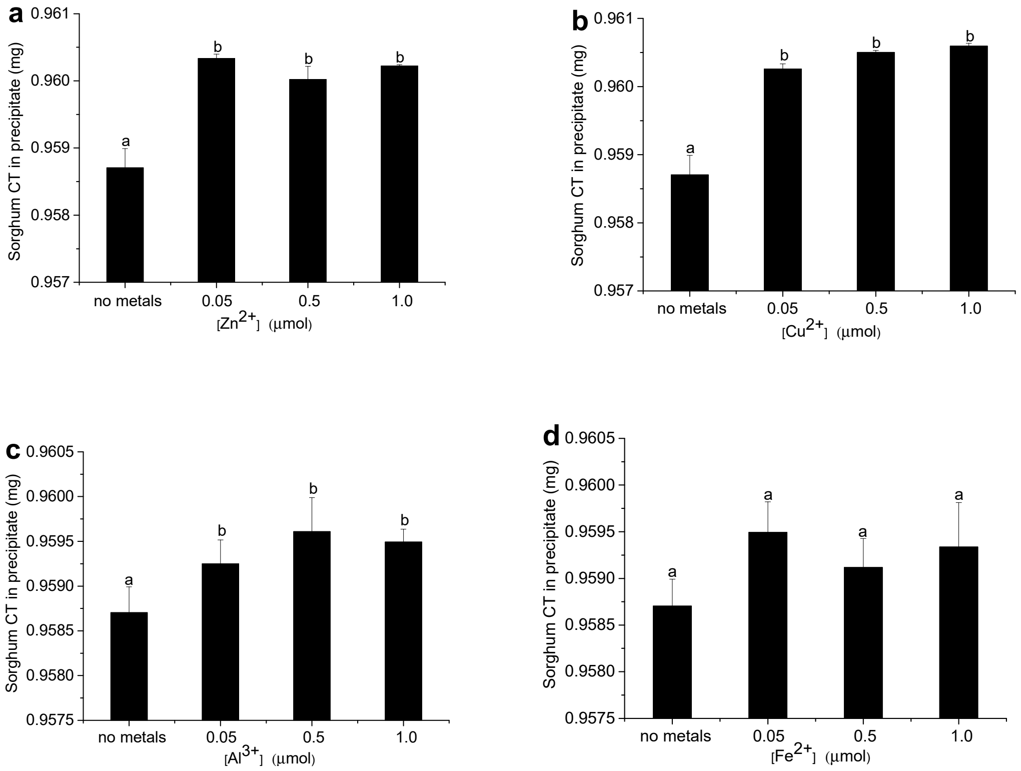

Supplemental Figure S1

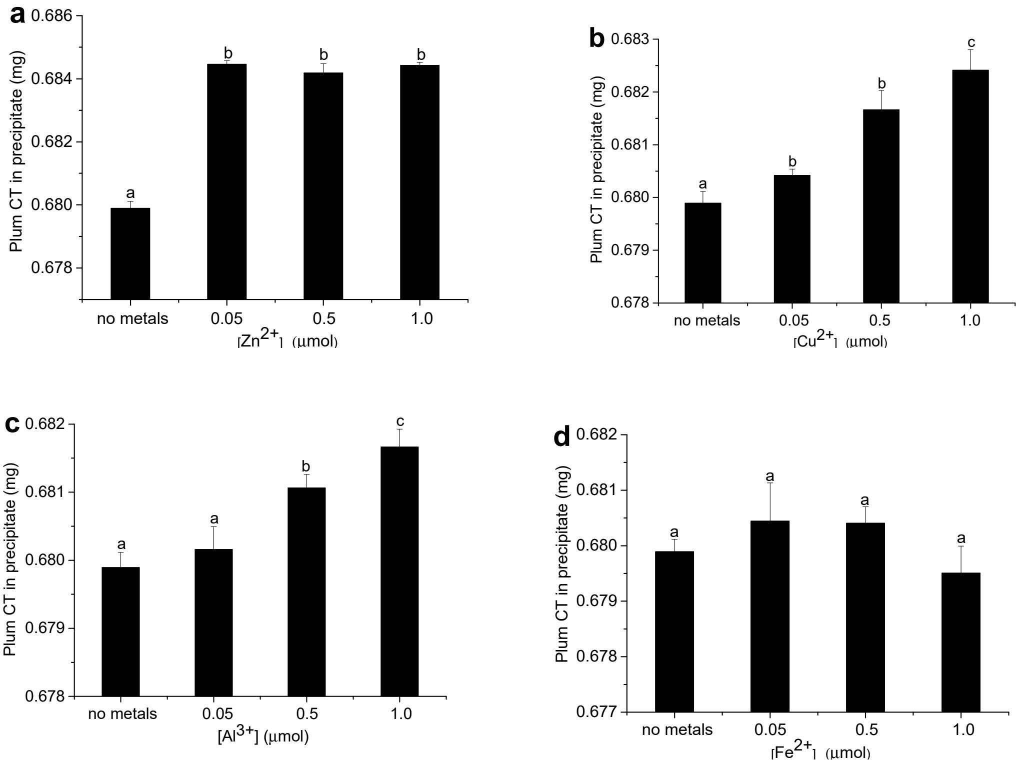

Supplemental Figure S2
